# Autoimmune Hepatobiliary Disease and Cryoglobulins in Peripheral Blood

**DOI:** 10.1002/ajh.27749

**Published:** 2025-07-03

**Authors:** Anna Shestakova, Anton V. Rets, Madhu Menon

**Affiliations:** ^1^ Department of Pathology University of Utah Salt Lake City Utah USA; ^2^ ARUP Laboratories Salt Lake City Utah USA

**Keywords:** cryoglobulin, cryoglobulins, cryoglobulins autoimmune hebatobiliary

## Abstract

Cryoglobulins in Autoimmune Hepatobiliary Disease.

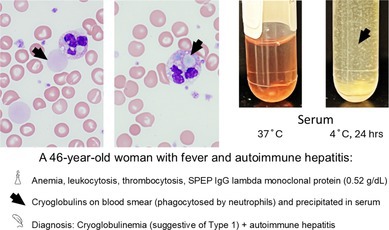

A 46‐year‐old woman presented with persistent fevers and abdominal pain. Laboratory evaluation revealed anemia (Hb 9.2 g/dL), leukocytosis (11 × 10^9^/L), and thrombocytosis (734 × 10^9^/L). Review of the peripheral blood smear demonstrated extracellular amorphous light‐blue and pink material, some of which was also phagocytosed by neutrophils, suggesting the presence of cryoglobulins (Figure 1A, B). Serum incubation at 4°C showed immunoglobulin precipitation primarily composed of IgG (22 mg/dL) with minor amounts of IgA and IgM (Figure 1C). Although serum protein electrophoresis revealed IgG lambda monoclonal protein (0.52 g/dL), a bone marrow biopsy showed no evidence of lymphoproliferative disorder.

**FIGURE 1:**
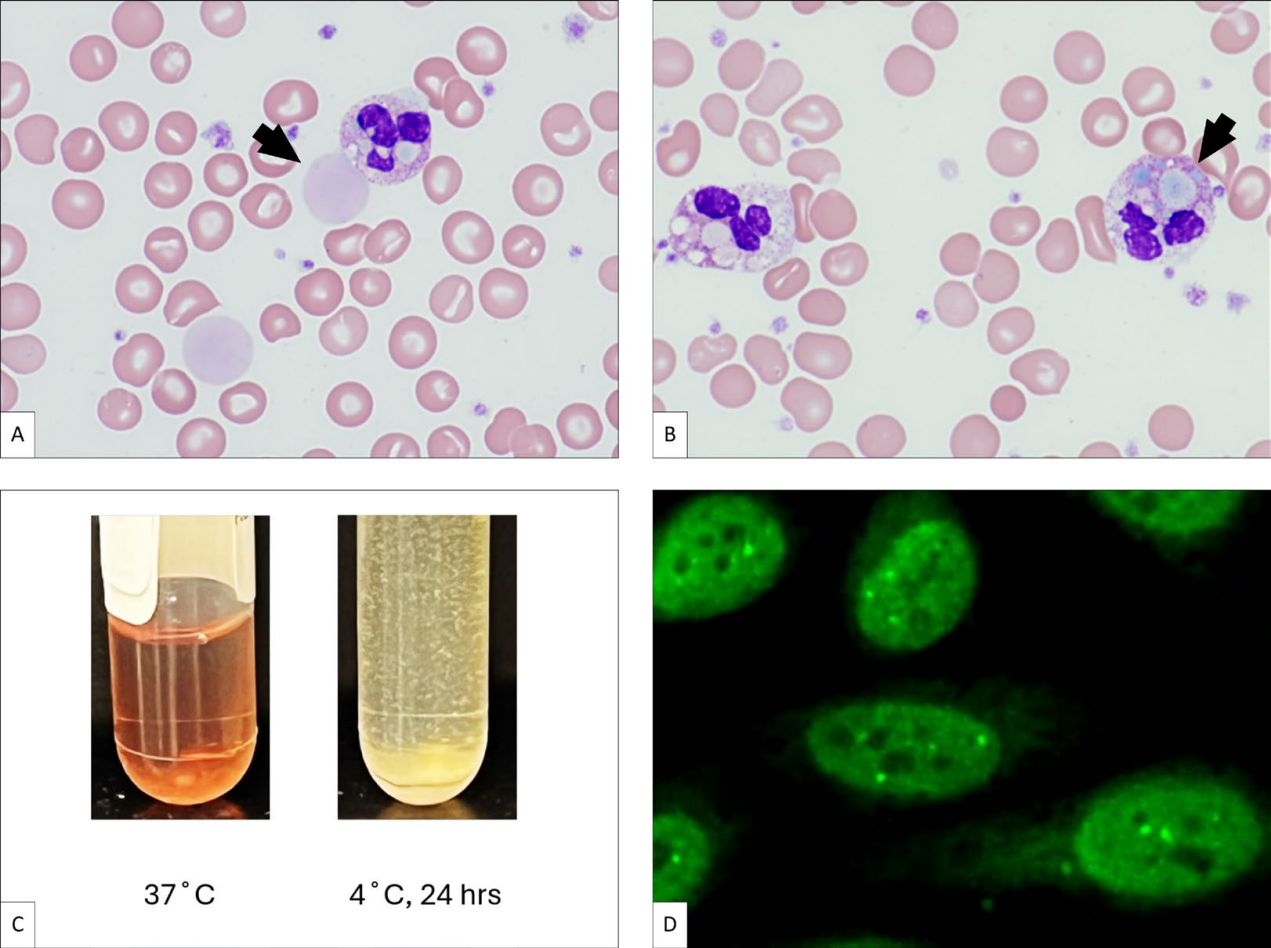
Cryoglobulins phagocytosed by neutrophils on peripheral blood smear. Amorphous light‐blue and pink material observed extracellularly (arrow, panel A) and within neutrophils (arrow, panel B), indicating phagocytosis of cryoglobulins. Positive cryoglobulin test (visible precipitate in the serum incubated at 4°C for 24 h) (panel C). Positive ANA indirect immunofluorescence (IFA), speckled pattern (bright green speckles, panel D).

Autoimmune hepatitis was diagnosed based on the liver biopsy, positive IgG antinuclear antibody (ANA) at a titer of 1:320 with a speckled pattern (Figure 1D), and the presence of F‐actin autoantibodies.

Cryoglobulins are immunoglobulins that precipitate when serum is cooled to 4°C and redissolve upon warming. Type I cryoglobulins consist of monoclonal immunoglobulins and can be associated with plasma cell neoplasm and non‐Hodgkin lymphoma, such as Waldenstrom's macroglobulinemia. Mixed cryoglobulinemia includes Type II (polyclonal IgG and monoclonal IgM with rheumatoid‐factor activity) and Type III (polyclonal IgG and/or IgM). These conditions can be associated with systemic autoimmune diseases and chronic infections, such as Hepatitis C virus [1]. Type I monoclonal cryoglobulins form stable complexes at lower temperatures, leading to mechanical vascular obstruction; in mixed cryoglobulinemia, immunoglobulins with rheumatoid‐factor activity induce complement‐mediated vascular inflammation and obstruction. Rarely, as in this case, cryoglobulins can be visualized within neutrophils.

## Ethics Statement

This is a retrospective review of data from a single patient that is performed according to the Institutional Review Board (IRB) University of Utah IRB_00077285.

## Conflicts of Interest

The authors declare no conflicts of interest.

## Data Availability

The data supporting these are available upon request from the corresponding author.
